# The relationship between depressive symptoms, general psychopathology, and well‐being in patients with major depressive disorder

**DOI:** 10.1002/jclp.23083

**Published:** 2020-11-14

**Authors:** Annelies Weijers, Sanne Rasing, Daan Creemers, Ad Vermulst, Arnt F. A. Schellekens, Gerben J. Westerhof

**Affiliations:** ^1^ GGZ Oost Brabant Boekel The Netherlands; ^2^ Behavioral Science Institute Radboud University Nijmegen The Netherlands; ^3^ Department of Psychiatry, Donders Institute for Brain, Cognition and Behavior Radboud University Medical Center Nijmegen The Netherlands; ^4^ Nijmegen Institute for Science Practitioners in Addiction (NISPA) Radboud University Nijmegen The Netherlands; ^5^ Department Psychology, Health, and Technology University of Twente Enschede The Netherlands

**Keywords:** depression, dual continua model, Mental Health Continuum‐Short Form, Routine Outcome Monitoring, well‐being

## Abstract

**Objective:**

In mental health care, treatment effects are commonly monitored by symptom severity measures. This study aimed to investigate the relationship between symptom severity and well‐being in the treatment of patients with major depressive disorder (MDD).

**Methods:**

Adult MDD outpatients (*n* = 77) were administered the Quick Inventory of Depressive Symptomatology—Self‐Report (QIDS‐SR), the Outcome Questionnaire (OQ‐45), and the Mental Health Continuum‐Short Form (MHC‐SF) before treatment and 6 months later.

**Results:**

Symptom severity correlated moderately with well‐being at baseline and strongly at follow‐up. Reliable change index scores showed improvement on the QIDS‐SR, OQ‐45, and MHC‐SF in 65%, 59%, and 40%, respectively. A quarter of patients improved in symptom severity but not well‐being (Inventory of Depressive Symptomatology—Self‐Report [IDS‐SR]: 25%; OQ‐45: 24%).

**Conclusion:**

Findings suggest that symptom severity and subjective well‐being are related, but distinct concepts. Several reasons for the stronger improvements in symptoms than in well‐being are discussed.

## 1 INTRODUCTION

Major depressive disorder (MDD) is the leading cause of disease burden worldwide (GBD 2015 Disease and Injury Incidence and Prevalence Collaborators, 2016; Rehm & Shield, 2019), with a lifetime prevalence of about 15%–18% worldwide (Kessler & Bromet, 2013; Kraus et al., 2019; Malhi & Mann, 2018). Available evidence‐based treatments for MDD are moderately effective, with about one‐third of patients responding to the initial treatment (Al‐Harbi, 2012; Maslej et al., 2020; Wittchen et al., 2011). Therefore, close monitoring of improvement is highly important. To this end, many treatment facilities implemented routine outcome monitoring (ROM) to systematically evaluate treatment effectiveness and to timely switch treatment.

ROM commonly consists of repeated self‐reported symptom severity levels (Goldfried & Wolfe, 1996). As such, disease severity and the pathology of the patient are the focus of treatment. However, the relevance of symptom reduction for the recovery process of patients with psychiatric disorders has been questioned, since mental health is considered much more than the absence of symptoms of psychopathology (Fava & Guidi, 2020; Keyes, 2002, 2005). The World Health Organization (WHO) views mental health as “a state of well‐being in which the individual realizes his or her own abilities, can cope with the normal stressors of life, can work productively and fruitfully, and is able to make a contribution to his or her community” (Galderisi et al., 2015).

The WHO definition of mental health has been adopted by the field of *positive psychology* as a three‐layered concept of mental well‐being, including emotional, psychological, and social well‐being (Lamers et al., 2012). Emotional well‐being is defined as the subjective experience of positive and negative feelings and the positive cognitive evaluation of one's own life (Diener, 1984; Diener et al., 1999). Psychological well‐being refers to individual functioning in the sense of self‐realization (Ryff, 1989; Ryff & Keyes, 1995; Ryff & Singer, 2008). It includes elements like autonomy, self‐acceptation, and meaning in life. Social well‐being refers to the personal perception of social role, including elements as feeling connected with society and the experience to be meaningful to society (Bohlmeijer et al., 2013, 2016).

Several studies indicate that symptom severity levels of psychiatric disorders and mental well‐being are two related, but distinct factors, the so‐called two‐continua model (Keyes, 2002, 2005; Tudor, 1996).

However, the two‐continua model has mainly been tested in community samples (Gilmour, 2014; Huppert & Whittington, 2003; Keyes, 2005; Lamers et al., 2011, 2012; Perugini et al., 2017; Westerhof & Keyes, 2010), students (Keyes et al., 2012; Renshaw & Cohen, 2014) and people with mild mental health problems (Trompetter et al., 2017). In community samples low to moderate correlations between psychopathology and well‐being were observed (*r* = −.35 to *r* = −.50). Moreover, most of these studies mainly applied general assessment tools of mental health instead of disorder‐specific measures, as commonly used in clinical practice.

Few studies did investigate the two‐continua model in clinical populations with severe mental health problems, including patients with severe depression. Franken et al. (2018) found a strong negative correlation between overall well‐being and psychopathology severity in outpatients with mood, anxiety, personality, and developmental disorders (complete sample: *r* = −.72; mood disorders: *r* = −.79). de Vos et al. (2018) examined the two‐continua model among 468 outpatients with eating disorders. They also observed a moderate to strong negative correlation between well‐being and psychopathology severity (*r* = −.73). A major limitation of these studies is that they did not apply a design with two measurements over time. As a result, information on the clinical applicability of the well‐being concept for ROM purposes is still limited.

A possible distinction between psychopathology and well‐being would have important clinical implications in mental health care services (Slade & Keating, 2010; van Erp Taalman Kip & Hutschemaekers, 2018). First, depression targeted treatments should ultimately result in increased well‐being (Bolier et al., 2013; Chakhssi et al., 2018; Sin & Lyubomirsky, 2009). Second, evidence emerges that increased well‐being might buffer against psychopathology and preventing relapse (Iasiello et al., 2019; Keyes et al., 2010; Schotanus‐Dijkstra et al., 2017; Trompetter et al., 2017). As a consequence, recovery should be measured both in terms of symptom reduction and increase in well‐being.

The current study aimed to investigate longitudinally the relationship between depression symptom severity, general psychopathology, and mental well‐being in a clinical sample. Based on previous studies, we hypothesized a strong negative correlation between depressive symptom severity, general psychopathology, and well‐being at the start of treatment and after 6 months in MDD patients. Furthermore, we explored the relationship between changes in depressive symptom severity measures, general psychopathology, and changes in well‐being during treatment in patients with MDD.

## 2 MATERIALS AND METHODS

### 2.1 Design

An observational cohort study was conducted, using Routine Outcome Monitoring data (collected as part of standard treatment evaluation). Participants provided written informed consent. The study protocol was approved by the regional ethical board (Faculty of Behavioral Sciences Ethics Committee at the University of Twente in the Netherlands).

### 2.2 Participants

Consecutive outpatients who applied for treatment for MDD (*n* = 77) were recruited at a regional mental health care facility (GGZ Oost Brabant, The Netherlands). Inclusion criteria were the primary diagnosis MDD (based on the Mini‐Neuropsychiatric Interview) and MDD as treatment focus. Primary diagnosis MDD was referred to as the patient's main mental health problem and causing most of the burden. Other inclusion criteria were age 18–65 years, good understanding of the Dutch language, and informed consent. Patients with psychosis were excluded.

Of the 125 approached patients, 77 were included in the study. Of the 48 patients that did not participate, 12 refused because they considered themselves too depressed to fill out questionnaires, 24 refused without a reason, five patients were excluded due to difficulties with the Dutch language, three had a treatment‐focus other than MDD, and four were referred to another mental health care organization.

Most participants were female (59.7%), with a partner, moderately educated, having paid employment, and suffering from severe MDD, according to DSM‐IV (see Table 1). Mean age was 41.9 years (standard deviation [*SD*] 11.4; range 22–64 years).

**Table 1:** Characteristics of the study sample

Characteristic	*n*	%
Age		
Mean	41.9	
Range	22–64	
*SD*	11.4	
Gender	31	40.3
Male	46	59.7
Female	31	60.3
Level of education^a^		
Low	25	32.5
MiLdle	31	40.3
High	21	27.3
Living situation		
Single	24	31.2
With partner	53	68.8
Employment		
Paid employment	46	59.7
Study	4	5.2
Unemployed	27	35.1
Severity of MDD		
Mild	0	0
Moderate	28	36.4
Severe	39	50.6
Unspecified	10	12.8
History of depression		
First episode	32	41.6
Recurrent depression	45	58.4

Abbreviation: MDD, major depressive disorder.

^a^ Low = primary school, lower vocational education; middle = secondary school, intermediate vocational education; high = higher vocational education, university.

At baseline 76 participants filled out instruments to measure depressive symptoms, 77 filled out a questionnaire to measure general psychopathology, and 76 filled out an instrument to measure well‐being. In total, 60 of 77 participants (76.9%) filled out the symptom severity questionnaires and the measure for well‐being at both measurements. Non‐completers were defined as participants who did not fill out questionnaires at the second measurement (6 months). These participants either ended therapy before the second measurement or decided to quit therapy. Fisher's exact test showed no significant group differences between completers and dropouts/non‐completers for the variables gender, marital status, education, work situation, recurrence of depression. Independent *t* tests indicated that the two groups were not significantly different at baseline levels for well‐being (see Supplementary Information). Non‐completers showed a significant lower level of depressive symptoms (Inventory of Depressive Symptomatology—Self‐Report [IDS‐SR] *M* = 35.2; *SD* = 17.5) at baseline compared to completers (IDS‐SR *M* = 45.7, *SD* = 11.3; *t*(74) = 2.9; *p* = .006).

### 2.3 Measurements

#### 2.3.1 Demographic information

Demographic information was collected during the intake interview, including the level of education, daily activities such as work or study, marital status, and history of previous depressive episodes (see Table 1).

#### 2.3.2 Depressive symptom severity

Depressive symptom severity was measured with the 30‐item IDS‐SR (Rush et al., 2003; Trivedi et al., 2004). The IDS‐SR was designed to assess the severity of depressive symptoms in line with the American Psychiatry Association Diagnostic and Statistical Manual of Mental Disorders (*Diagnostical and statistical manual of mental disorders (DSM IV‐TR)*, 2000) 4th edition (2003). Respondents rate the frequency of depressive symptoms in the past week using a four‐point Likert scale ranging from 0 to 3. The IDS‐SR is sensitive to change, with pharmacotherapy and psychotherapy, making it useful for both research and clinical purposes. The psychometric properties of the IDS‐SR have been established in various samples. Internal consistency was high for the IDS‐SR (Cronbach's *α* = .86 to .94) (Rush et al., 2003; Trivedi et al., 2004).

#### 2.3.3 General psychopathology severity

The Outcome Questionnaire‐45 (OQ‐45) was administered to measure general psychopathology. The OQ‐45 is a 45‐item self‐report measure, developed for the assessment of treatment outcomes in a therapeutic setting (Jong et al., 2008). It was designed to assess three main areas of functioning: symptomatic distress/general psychopathology (including anxiety and depression: 25 items), interpersonal problems (friendship and family relations: 11 items), and social role performance (work adjustment and quality of life: 9 items). Respondents rate the frequency of symptoms in the past week using a five‐point Likert scale ranging from 0 (“*never*”) to 4 (“*almost always*”). Higher scores indicate more psychological distress. The psychometric properties of the OQ‐45 appear to be good across a variety of cultures (Lo Coco et al., 2008). Test–retest reliability of the OQ‐45 among psychiatric patients varied between *α* = .70 and *α* = .83 (Jong et al., 2008). Internal consistency for the total test‐scores among psychiatric patients was high (*α* = .93; de Jong et al., 2008) as well as for the subscales general psychopathology (*α* = .84) and interpersonal relations (*α* = .81) and moderate for social roles (*α* = .69).

#### 2.3.4 Well‐being

Well‐being was measured by the Dutch version of the Mental Health Continuum‐Short Form (MHC‐SF; Keyes et al., 2008; Lamers et al., 2011). The MHC‐SF is based on a longer self‐report questionnaire (Keyes, 2002). The instrument comprises 14 items, representing various feelings of well‐being. Respondents rate the frequency of every feeling in the past month on a 6‐point Likert scale from 0 (“*never*”) to 5 (“*every day*”). The MHC‐SF contains three items of emotional well‐being, six items of psychological well‐being, and five items of social well‐being, with each psychological and social well‐being item representing one dimension. A higher average score (0–5) indicates a higher level of well‐being (Lamers et al., 2011). The MHC‐SF has shown good psychometric properties in the Dutch population (Franken et al., 2018; Lamers et al., 2011, 2012) with high internal reliability for the total score (*α* = .89), as well as for the subscales of emotional well‐being (*α* = .83) and psychological well‐being (*α* = .83), and adequate reliability for the subscale social well‐being (*α* = .74). The internal consistency for the total scale of the MHC‐SF in the study of Franken et al. (2018) among a clinical population was high (*α* = .92), as well as for the subscales emotional well‐being (*α* = .88) and psychological well‐being (*α* = .85). For social well‐being, the internal consistency was acceptable (*α* = .77).

### 2.4 Procedure

At intake, the MINI (Mini‐International Neuropsychiatric Interview) was administered by a licensed psychiatrist, psychologist, or nurse specialist to classify symptoms according to the DSM‐IV. Patients who met the inclusion criteria (primary diagnosis depression and age 18–65 years), were informed at intake by their mental health professional about the study. If necessary, more information about the study was provided by the researcher or a research assistant. After signing an informed consent, participants were administered self‐report questionnaires at the start of treatment and 6 months later. Participants received treatment as usual: pharmacotherapy and/or psychotherapy (cognitive behavioral therapy or interpersonal therapy), based on patient preference and guideline recommendations through a shared decision‐making process. Data were collected between June 2017 and May 2019 and anonymized before data analysis.

### 2.5 Statistical analyses

Analyses were performed in Statistical Package of the Social Sciences (SPSS 24) and Mplus version 7.2 (Muthen & Muthen, 1998‐2012). Pearson correlations were calculated between depressive symptom severity/general psychopathology and well‐being at baseline and follow‐up. Correlations were interpreted as follows (Santrock, 2007): very low, *r* = 0–.20; low, *r* = .21–.40; moderate, *r* = .41–.60; moderately high: *r* = .61–.80; high: *r* = .81–.90, and very high: *r* = .91–1.0.

To examine individual progress over time for depression symptoms/general psychopathology and well‐being Reliable Change Indices (RCI) were computed for each patient by dividing the difference of the scores before treatment and 6 months follow‐up by the standard error of this difference according to Jacobson and Truax (1992). RCI's are standardized scores assumed to have a normal distribution probability distribution. Because we expect progress over time, patients with RCI‐scores > 1.645 are classified as significantly improved with *p* = .05, patients with −1.645 < RCI‐scores < 1.645 are classified as not significantly improved with *p* = .90, and patients with RCI‐scores < −1.645 as significantly deteriorated with *p* = .05. The standard error of differences in the denominator of RCI are based on the *SD*s and reliabilities of the scales. *SD*s and reliabilities for the IDS‐R are based on the study of Schulte‐van Maaren et al. (2013), for the Q‐45 the study of De Jong et al. (2018), and for the MHC‐SF the study of Franken et al. (2018). The three studies report reliability and validity studies in the Netherlands for each of the three measuring instruments, respectively. We used the *SD* and reliabilities of the patient populations reported in these studies. Mostly internal consistencies are reported, therefore we used Cronbach's *α* as a reliability measure.

The classifications of patients (improved, not improved, and deteriorated) were cross‐classified: depressive symptoms with well‐being and general psychopathology with well‐being. With Fisher's exact test it was tested whether the classification of well‐being was associated with the classification in depressive symptoms and general psychopathology. The classifications are ordinal, so we used Kendall's Tau‐b or Tau‐c to measure the association between both classifications (both measures are corrected for ties with Tau‐b for square tables and Tau‐c for rectangular tables).

## 3 RESULTS

### 3.1 Relation between psychopathology and well‐being

Mean levels and *SD*s of the IDS‐SR, the OQ‐45, and the MHC‐SF and their subscales at baseline and after 6 months are presented in Table 2. With *t* tests for dependent samples, we tested whether psychopathology decreased and well‐being increased. Effects sizes (Cohen's *d*) are calculated (*d* = 0.2 is a small effect, *d* = 0.5 a medium effect, and *d* = 0.8 a large effect). In Table 2 all measures of psychopathology show significant decreases over time with large effect sizes except for interpersonal relations (small to medium effect size). The measure of well‐being shows significant increases over time with medium to large effect sizes except for social well‐being with a medium effect size.

**Table 2:** Severity of psychopathology and subjective levels of well‐being and at baseline (T1) and after 6 months (T2)

	Baseline (T1)			After 6 months (T2)					
	*N*	*M*	*SD*	*N*	*M*	*SD*	*t*	*p*	*d*
Psychopathology									
IDS‐SR	76	43.6	13.3	61	31.6	15.7	8.06	.000	1.03
OQ‐45 general psychopathology	77	60.1	16.6	61	47.1	18.7	7.21	.000	0.93
OQ‐45 Interpersonal relations	77	18.4	76.9	61	15.5	7.5	3.05	.003	0.39
OQ‐45 social roles	77	16.1	5.6	61	12.6	5.9	6.07	.000	0.78
OQ‐45 Total	77	94.5	24.5	61	75.2	28.7	6.86	.000	0.89
Well‐being									
MHC‐SF emotional well‐being	76	1.51	1.29	60	2.30	1.39	−4.64	.000	0.60
MHC‐SF psychological well‐being	76	1.66	1.06	60	2.28	1.24	−5.30	.000	0.68
MHC‐SF social well‐being	76	1.51	1.08	60	1.89	1.04	−3.84	.000	0.50
MHC‐SF total well‐being	76	1.57	0.97	60	2.15	1.11	−5.77	.000	0.75

Abbreviations: IDS‐SR, Inventory of Depressive Symptomatology—Self‐report; MHC‐SF, Mental Health Continuum‐Short Form; OQ, Outcome Questionnaire.

Correlations between the IDS‐SR, the OQ‐45, and the MHC‐SF and their subscales at baseline are presented in Table 3. At baseline, moderate negative correlations were found between depressive symptom severity and well‐being (*r* = −.56; *p* < .001) and high moderate correlations between general psychopathology and well‐being (*r* = −.66; *p* < .001). Moderate negative correlation coefficients were found for the subscales of the MHC‐SF and the subscales of the OQ‐45 (*r* = −.42 to −.62), see Table 3.

**Table 3:** Correlations between depressive symptom severity (IDS‐SR), general psychopathology (OQ‐45), and well‐being (MHC‐SF) at baseline

Psychopathology at baseline (T1)	Psychopathology					Well‐being MHC‐SF at baseline (T1; *N* = 76)			
	IDS‐SR	OQ‐45 GP	OQ‐45 IR	OQ‐45 SR	OQ‐45 Total	Emotional well‐being	Psychological well‐being	Social well‐being	Total well‐being
IDS‐SR (*N* = 76)	1	0.86**	0.44**	0.53**	0.82**	−0.54**	−0.53**	−0.39**	−0.56**
OQ‐45 GP	0.86**	1	0.55**	0.56**	0.95**	−0.62**	−0.56**	−0.43**	−0.60**
OQ‐45 IR	0.44**	0.55**	1	0.44**	0.74**	−0.47**	−0.58**	−0.42**	−0.56**
OQ‐45 SR	0.53**	0.56**	0.44**	1	0.72**	−0.35**	−0.37**	−0.42**	−0.45**
OQ‐45 Total (*N* = 77)	0.82**	0.95**	0.74**	0.72**	1	−0.62**	−0.62**	−0.49**	−0.66**

Abbreviations: GP, general psychopathology; IDS‐SR, Inventory of Depressive Symptomatology—Self‐report; IR, interpersonal relations; MHC‐SF, Mental Health Continuum‐Short Form; OQ, Outcome Questionnaire; SR, social roles.

** *p* < .01, two‐tailed.

At the second measurement (after 6 months) strong negative correlations were found between depressive symptom severity and well‐being (*r* = −.79; *p* < .001) and between general psychopathology and well‐being (*r* = −.80; *p* < .001; Table 4). Moderate to strong negative correlation coefficients were found for the subscales of the OQ‐45 and the subscales of the MHC‐SF (*r* = −.47 to −.77).

**Table 4:** Correlations between depressive symptom severity, general psychopathology, and well‐being after 6 months (T2)

Psychopathology after 6 months (T2)	Psychopathology					Well‐being MHC‐SF after 6 months (T2; *N* = 60)			
	IDS‐SR	OQ‐45 GP	OQ‐45 IR	OQ‐45 SR	OQ‐45 Total	Emotional well‐being	Psychological well‐being	Social well‐being	Total well‐being
IDS‐SR (*N* = 61)	1	0.84**	0.52**	0.62**	0.84**	−0.82**	−0.72**	−0.66**	−0.79**
OQ‐45 GP	0.89**	1	0.66**	0.68**	0.96**	−0.75**	−0.73**	−0.70**	−0.79**
OQ‐45 IR	0.52**	0.66**	1	0.56**	0.81**	−0.47**	−0.67**	−0.53**	−0.62**
OQ‐45 SR	0.62**	0.68**	0.44**	1	0.80**	−0.48**	−0.60**	−0.56**	−0.60**
OQ‐45 Total (*N* = 61)	0.84**	0.96**	0.81**	0.80**	1	−0.70**	−0.77**	−0.70**	−0.80**

Abbreviations: GP, general psychopathology; IDS‐SR, Inventory of Depressive Symptomatology—Self‐report; IR, interpersonal relations; MHC‐SF, Mental Health Continuum‐Short Form; OQ, Outcome Questionnaire; SR, social roles.

** *p* < .01, two‐tailed.

Pearson's correlations between depressive symptoms, general psychopathology, and well‐being at baseline showed similar correlations for completers only (IDS‐SR and MHC‐SF: *r* = .51; OQ‐45 and MHC‐SF: *r* = .64), as compared to the whole baseline sample. Scatter plots (see Supplementary File 1) revealed that the relative increase in correlation was mainly driven by some patients with a high level of symptom severity and a moderate to a high level of well‐being at baseline, who improved in terms of symptom severity at follow‐up.

### 3.2 Changes in psychopathology and well‐being

In Table 5, individual change in psychopathology and well‐being in terms of their classification of the reliable change index (improved, not improved, and deteriorated) is given including cross‐classifications of depressive symptoms with well‐being and general psychopathology with well‐being. For both classifications, the associations were negative (increasing reliable change in well‐being means a decrease in the reliable change in depressive symptoms and general psychopathology) and significant (the *p* values of Fisher's exact test were ≤.001) with moderate levels of association. Overall, 59.3% of the participants decreased on depressive symptoms, 35.6% did not change, and 5.1% increased. For general psychopathology, 65% decreased, 30% did not change, and 5% increased. In all, 40% of the participants increased on well‐being, 60% did not change, and nobody decreased.

**Table 5:** Reliable change in depressive symptoms and/or well‐being from baseline to follow‐up—Reference group psychiatric patients

	Depressive symptoms (IDS‐SR)				General psychopathology (OQ‐45)			
	Increase	No change	Decrease	Total	Increase	No change	Decrease	Total
Mental health (MHC‐SF)								
Increase (%)	0	5	35.6	40.7	0	0	40	40
No change (%)	5.1	30.5	23.7	59.3	5	30	25	60
Decrease (%)	0	0	0	0	0	0	0	0
Total (%)	5.1	35.6	59.3	100	5	30	65	100

*Note:* Fisher's exact test: *p* = .000, Tau‐c = −0.56 (IDS‐SR–MHC‐SF); Fisher's exact test: *p* = .001, Tau‐c = −0.47 (OQ‐45–MHC‐SF).

Abbreviations: IDS‐SR, Inventory of Depressive Symptomatology—Self‐report; MHC‐SF, Mental Health Continuum‐Short Form; OQ, Outcome Questionnaire.

Furthermore, about a quarter of the patients showed a decrease in depressive symptoms (IDS‐SR 25%) and general psychopathology (OQ‐45 23.7%), but no changes on well‐being. Only few patients showed an increase on well‐being but no changes on psychopathology (5% OQ‐45; 0% IDS‐SR). Deterioration was observed in 5% on both IDS‐SR and OQ‐45, none deteriorated on the MHC‐SF.

Cross‐tabulation of classifications of reliable change indices of the subscales and total scales of MHC‐SF with IDS‐SR and OQ‐45 (Table S3) showed significant negative associations (with *p* values of Fisher's exact test between .000 and .008 and mostly moderate levels of association) with the exception of the subscale social well‐being with the (sub)scales of IDS‐SR, OQ‐45, and emotional well‐being with social roles and of total well‐being with social roles.

## 4 DISCUSSION

The aim of this study was to analyze prospectively the relationship between depressive symptom severity, general psychopathology, and well‐being within a group of MDD‐patients in treatment. Moderate negative correlations were found between depressive symptom severity/general psychopathology and well‐being at baseline, and strong negative correlations at follow‐up. Most patients showed a decrease in symptom severity levels (60%–65%), whereas less than half (40%) showed an increase in well‐being. Only about a quarter of the study population showed symptom reduction (IDS‐SR, OQ‐45) as well as an increase in well‐being (MHC‐SF).

The observed moderate correlations between symptom severity and well‐being at baseline were stronger than those previously observed in the general population (Guo et al., 2015; Keyes, 2005; Lamers et al., 2011; Perugini et al., 2017), comparable to findings in people suffering mild depressive symptoms (Huppert & Whittington, 2003; Moeenizadeh & Salagme, 2010; Renshaw & Cohen, 2014; Schotanus‐dijkstra et al., 2017), and somewhat smaller than those observed in clinical studies. However, symptom severity at baseline was stronger in this study than in other clinical studies (de Vos et al., 2018; Franken et al., 2018; Trompetter et al., 2017; van Erp Taalman Kip & Hutschemaekers, 2018). Furthermore, the stronger correlations at follow‐up were comparable to other cross‐sectional clinical studies (de Vos et al., 2018; Franken et al., 2018; van Erp Taalman Kip & Hutschemaekers, 2018).

This variance in the strength of the correlation between symptom severity and levels of well‐being might be explained by an inverted U‐shape relationship. In the absence of psychopathology (in the general population) the link between symptom severity and well‐being might be relatively weak since other factors are more relevant for well‐being than symptoms of psychopathology. In people with moderate depressive symptoms, the correlation between depression severity and well‐being might become stronger.

Whereas some studies suggest that people with severe depression might not be able to distinguish between mental illness and mental health (Haeyen et al., 2017; Lukat et al., 2016; van Erp Taalman Kip & Hutschemaekers, 2018), we observed in our study that despite high levels of depression, well‐being was relatively preserved in some patients, reducing the strength of the correlation with symptom severity (Supplementary File 3, scatterplot). As observed in the current study, in patients who recover from their depression, the relation between symptom severity and well‐being might increase again. This further supports the idea that though related, well‐being is conceptually different from disease severity, in line with the two‐continua model.

While most participants improved on levels of symptom severity (IDS‐SR: 65%; OQ‐45: 59%), less than half improved on well‐being (40%). These findings are in line with findings in the study of Trompetter et al. (2017), showing that only 36% of the patients showed improvement in both depressive symptoms *and* well‐being, while 64% improved in one outcome (mainly symptom severity) but not in the other. On the one hand these data might support the two‐continua model that psychopathology and well‐being are related, but distinct concepts (Bohlmeijer et al., 2016; Keyes, 2005; Keyes et al., 2008; Lamers et al., 2011). On the other hand, the measures of psychopathology used here (IDS‐SR and OQ‐45) might be more sensitive to change than the measure of well‐being (MHC‐SF). Either way, symptom severity measures (IDS‐SR and OQ‐45) might be more sensitive measures on the short term for follow‐up of treatment effectiveness than well‐being as indexed by MHC‐SF.

Another explanation for the observed differences in changes in symptom severity measures and our measure of well‐being might be the nature and focus of treatment. Several studies indicate that patients define “good therapy outcome” as clustered around four themes: establishing new ways of relating to others; less symptomatic distress, or changes in behavioral patterns contributing to suffering; better self‐understanding and insight; and accepting and valuing oneself (Binder et al., 2010; Cuijpers, 2020). The applied treatments in our study (both pharmacotherapy and psychotherapy) are mainly symptom focused therapies, thus mainly leading to symptom reductions. Well‐being targeted therapies might have more impact on subjective well‐being (Fava et al., 2017; Schotanus‐Dijkstra et al., 2016). A few meta‐analyses provided evidence that positive psychology interventions (PPIs) are effective in improving well‐being as well as in alleviating common psychological symptoms, including depression, in clinical samples with psychiatric and somatic disorders (Bolier et al., 2013; Chakhssi et al., 2018; Sin & Lyubomirsky, 2009). A critical examination of these meta‐analyses by White et al. (2019) revealed that taken into account small sample size bias, the effect of PPIs on well‐being were small but significant (approximately *r* = .10), whereas the effect of PPIs on depression were variable, dependent on outliers, and generally not statistically significant. Future PPI research needs to focus on increasing sample sizes to investigate whether the nature of treatment accounts for changes on depressive symptom severity, general psychopathology, and well‐being.

Finally, the differences in changes in depressive symptom severity, general psychopathology, and well‐being might be explained by the duration of follow‐up in relation to the speed of change in both concepts. One aspect might be related to the time window of inquiry. The IDS‐SR and the OQ‐45 ask about the past week, whereas the MHC‐SF asks about the past month. Well‐being might thus be less sensitive to change, given the longer time window of inquiry. Alternatively, improvement in well‐being might indeed require more time than reduction in symptom severity. Since well‐being measures a broad variety in aspects of living, including individual functioning, self‐realization, autonomy, self‐acceptance, and fulfillment of social roles (Bohlmeijer et al., 2016; Lamers et al., 2012), this might take more time to change than symptom severity levels related only to psychopathology. One previous study indeed showed that in people with mild depression, the strongest improvements in well‐being were found 12 months after ending the intervention (Schotanus‐Dijkstra et al., 2017). It could thus be speculated that well‐being might be less sensitive to short‐term change than symptom severity, which should be further explored in future studies.

Another explanation for the observed lag between symptom improvement and increased well‐being might well be that well‐being is more likely to increase, only after patients have (partly) recovered from their depression. Some researchers even suggest that first symptoms should be treated to be continued by PPIs to strengthen resilience and to prevent relapse (e.g., Fava et al., 2017).

The current findings should be seen in light of several study limitations. The first limitation might be the duration of the follow‐up measurement after 6 months. Since improvement in well‐being might take more time than improvement in depressive symptoms, future research should include long‐term follow‐up (for instance, for a year) to better understand changes in well‐being and psychopathology over time.

A second limitation of the current study is that participants were collected in one regional mental health center, with a catchment area of 250,000 persons. Although the sample in this study is comparable to other studies on patients with MDD in terms of age, gender, and symptom severity (Franken et al., 2018; Trompetter et al., 2017), any effect of selection bias cannot fully be ruled out.

Furthermore, 35% of the study population were “jobless.” This category consisted of a heterogeneous group of people, who consciously chose to stay at home (e.g, take care of the kids) or who lost their job (e.g., due to depression). Unemployment might have affected the relationship between depression severity and well‐being.

A fourth limitation of this study is addressed to the potential effect of comorbidity on treatment outcomes. For it was not an explicit research question, we did not include comorbidity as a separate variable in our study, though it might be relevant to include this aspect in future research.

While this is an observational study and our aim was to examine the relation between depressive symptoms/psychopathology and well‐being, the small study sample did not allow us to split the cohort for the type of treatment patients received. The impact of pharmacotherapy, psychotherapy, or a combination of both on mental illness and well‐being together might be a potential area for future research.

To continue, we applied only one well‐being measure in the current study, though several instruments are available. We chose the MHC‐SF because it had the best psychometric properties compared to other measures of well‐being, was validated for both Dutch general (Lamers et al., 2012) and clinical populations (Franken et al., 2018), consisted of different domains of well‐being, and had a limited number of questions, limiting the burden for patients. Future studies might explore how to best measure well‐being, for example, the Psychological Well‐Being Scale (Brandel et al., 2017; van Dierendonck, 2005; Fava et al., 1998; Ryff & Keyes, 1995), the Flourishing Scale (Diener, 1984; Schotanus‐Dijkstra et al., 2016), or the Warwick Edinburgh Mental Well‐Being Scale (Tennant et al., 2007) considering duration period of restoring well‐being and sensitivity to change over time.

Furthermore, the difference in time span the questionnaires query about, might be a potential confound. The IDS‐SR and OQ‐45 ask about the past week, whereas the MHC‐SF asks about the past month. Although the measure of depressive symptoms assesses frequency per week and the measure of well‐being frequency per month, pilot studies on the MHC‐SF showed that its correlation with depressive symptoms did not differ significantly when participants were asked for the past week instead of the past month (Westerhof & Keyes, 2008). Besides, epidemiological studies show that more than half of the general population with MDD recovers within 3 months (Spijker et al., 2002; Ten Have et al., 2017). This further dilutes any potential confounding effects of the differences in the assessment window between the outcome measures.

Finally, we administered the MINI only at the first measurement and used continuous outcome data. Some studies suggest that the MHC‐SF performs differently when using the categorical versus the continuous outcome data (Keyes, 2002; Schotanus‐Dijkstra et al., 2017). Future research might measure both types of outcome data and administer the MINI at follow‐up to investigate the relation between categorical changes in psychopathology and changes in well‐being.

## 5 CONCLUSION

Taken together, these results suggest that the severity of depression/general psychopathology and subjective well‐being are related, but distinct concepts, in line with the two‐continua model. Since symptom severity levels seemed more sensitive to change during treatment than subjective well‐being, psychopathology measures might be more useful than well‐being measures for the purpose of short‐term treatment evaluation to timely switch treatment. Although well‐being might seem less sensitive to change on the short term, long‐term monitoring of well‐being might be still be relevant, since well‐being entails a broader concept of recovery than symptom reduction, is considered important by patients, and has been associated with reduced risk of relapse.

## CONFLICT OF INTERESTS

The authors declare that there are no conflict of interests.

## Supporting information

Supporting information.
