# Supplementary material for: The relationship between depressive symptoms, general psychopathology, and well‐being in patients with major depressive disorder
Source: J Clin Psychol. 2020 Nov 14;77(6):1472–86. doi: 10.1002/jclp.23083 (PMC8246916; doi:10.1002/jclp.23083)
Supplement: Supplementary file 1 — Supporting information. [file JCLP-77-1472-s001.docx]

**Supplementary file 1: correlations psychopathology/depressive symptoms and well-being at baseline and follow-up (after 6 months)**

Table 1. correlations psychopathology/depressive symptoms and well-being at baseline

| baseline (T1)  N=77 | **Psychopathology (N=77)** | | | | | **Wellbeing MHC-SF at baseline (T1; N=77)** | | | |
| --- | --- | --- | --- | --- | --- | --- | --- | --- | --- |
|  | OQ-45 GP  (mean= 60.1; SD=16,3) | OQ-45 IR  (mean=18.4; SD=6,9) | OQ-45 SR  (mean=16,1; SD=5,6) | OQ-45 total  (mean=94,5; SD=24,5) | IDS-SR  (mean=43,6; SD=13,3) | EWB  (mean=1,5  SD=1,3) | PWB  (mean=1,7; SD=1,1) | SWB  (mean=1,5;  SD=1,1) | TWB (mean=1,6  SD=.98) |
| OQ-45 GP  (Mean= 60,1; SD=16,3) | 1 | .57** | .55** | .95** | .86** | -.62** | -.56** | -.43** | -.60** |
| OQ-45 IR (Mean=18,4; SD=6,9) | .57** | 1 | .43** | .56** | .44** | -.47** | -.58** | -.42** | -.56** |
| OQ-45 SR  (mean=16,1; SD=5,6) | .55** | .43** | 1 | .71** | .53** | -.35** | -.37** | -.42** | -.45** |
| OQ-45 Total (N=77)  (mean=94,5; SD=24,5) | .95** | .75** | .71** | 1 | .82** | -.62** | -.62** | -.49** | -.66** |
| IDS-SR (N=76)  (mean=43,6; SD=13,3) | .86** | .44** | .53** | .82** | 1 | -.54** | -.53** | -.39** | -.56** |
| EWB  (mean=1,5; SD=1,3) | -.62** | -.47** | -.35** | -.62** | -.54** | 1 | .58** | .57** | .74** |
| PWB  (mean=1,7; SD=1,1) | -.56** | -.58** | -.37** | -.62** | -.53** | .58** | 1 | .68** | .91** |
| SWB  (mean=1,5; SD=1,1) | -.43** | -.42** | -.42** | -.49** | -.39** | .57** | .68** | 1 | .88** |
| Total well-being  (mean=1,6; SD=.98) | -.60** | -.56** | -.45** | -.66** | -.56** | .74** | .91** | .88** | 1 |

Note **p < 0.01, two-tailed; GP: general psychopathology; IR: interpersonal relations; SR: social roles; EWB: emotional well-being; PWB: psychological well-being; SWB: social well-being; TWB: total well-being

Table 2. correlations psychopathology/depressive symptoms and well-being at follow-up (6 months)

| Follow-up T2 | **Psychopathology (N=61)** | | | | | **Wellbeing MHC-SF at baseline (N=60)** | | | |
| --- | --- | --- | --- | --- | --- | --- | --- | --- | --- |
|  | OQ-45 GP  (Mean= 60,1; SD=16,3) | OQ-45 IR  (Mean=18,4; SD=6,9) | OQ-45 SR  (mean=16,1; SD=5,6) | OQ-45Total  (mean=94,5; SD=24,5) | IDS-SR  (mean=43,6; SD=13,3) | EWB  (mean=1,5  SD=1,3) | PWB  (mean=1,7; SD=1,1) | SWB  (mean=1,5;  SD=1,1) | TWB  (mean=1,6  SD=.98) |
| OQ-45 GP  (Mean= 60,1;SD=16,3) | 1 | .66** | .68** | .96** | .89** | -.75** | -.74** | -.70** | -.79** |
| OQ-45 IR  (Mean=18,4; SD=6,9) | .66** | 1 | .56** | .81** | .52** | -.47** | -.67** | -.53** | -.62** |
| OQ-45 SR  (mean=16,1; SD=5,6) | .68** | .56* | 1 | .80** | .862* | -.48** | -.60** | -.56** | -.60** |
| OQ-45 Total (N=77)  (mean=94,5; SD=24,5) | .96** | .81** | .47** | 1 | .84** | -.71** | -.77** | -.71** | -.79** |
| IDS-SR (N=76)  (mean=43,6; SD=13,3) | .89** | .52** | .67** | .84** | 1 | -.82** | -.72** | -.66** | -.79** |
| EWB  (mean=1,5; SD=1,3) | -.75** | -.53** | -.48** | -.71** | -.82** | 1 | .79** | .71** | .89** |
| PWB  (mean=1,7; SD=1,1) | -.73** | -.62** | -.60** | -.77** | -.72** | .79** | 1 | .76** | .95** |
| SWB  (mean=1,5; SD=1,1) | -*70* | -.53** | -.56** | -.71** | -.66** | .71** | .76** | 1 | .89** |
| Total well-being  (mean=1,6; SD=.98) | -*79* | -.62** | -.60** | -.79** | -.79** | .89** | .95** | .89** | 1 |

Note **p < 0.01, two-tailed; GP: general psychopathology; IR: interpersonal relations; SR: social roles; EWB: emotional well-being; PWB: psychological well-being; SWB: social well-being; TWB: total well-being

**Supplementary file 2: cross-table reliable change (sub)scales at baseline and follow up**

Table 3. reliable change in depressive symptoms and/or wellbeing from baseline to follow-up. Reference group psychiatric patients

|  | | | | Well-being (MHC-SF) | | | | | | | | | | | | | | | |
| --- | --- | --- | --- | --- | --- | --- | --- | --- | --- | --- | --- | --- | --- | --- | --- | --- | --- | --- | --- |
|  |  |  |  | Emotional well-being | | | | Psychological well-being | | | | Social well-being | | | | Total well-being | | | |
|  |  |  |  | i | nc | d | t | i | nc | d | t | i | nc | d | t | i | nc | d | t |
| Psy cho pa tho logy | OQ-45  General  Psycho- patho-logy | i | N  % | 0  0 | 3  5,1 | 0  0 | 3**a  5.1 | 0  0 | 3  5.1 | 0  0 | 3**f  5.1 | 0  0 | 3  5.1 | 0  0 | 3 k  5.1 | 0  0 | 3  5.1 | 0  0 | 3**p  5.1 |
|  |  | nc | N  % | 1  1.7 | 20  33,9 | 1  1.7 | 22  37.3 | 1  1.7 | 21  35.6 | 0  0 | 22  37.3 | 2  3.4 | 20  33.9 | 0  0 | 22  37.3 | 3  5.1 | 19  32.2 | 0  0 | 22  37.3 |
|  |  | d | N  % | 21  35.6 | 13  22 | 0  0 | 34  57.6 | 15  25.4 | 19  32.2 | 0  0 | 34  57.6 | 11  18.6 | 22  37.3 | 1  1.7 | 34  57.6 | 21  35.6 | 13  22 | 0  0 | 34  57.6 |
|  |  | t | N  % | 22  37.3 | 36  61 | 1  1.7 | 59  100 | 16  27.1 | 43  72.9 | 0  0 | 59  100 | 13  22 | 45  76.3 | 1  1.7 | 59  100 | 24  40.7 | 35  59.3 | 0  0 | 59  100 |
|  | OQ-45  Inter-personal  Relations | i | N  % | 0  0 | 3  5.1 | 0  0 | 3**b  5.2 | 0  0 | 3  5.1 | 0  0 | 3**g  5.1 | 0  0 | 3  5.1 | 0  0 | 3 l  5.1 | 0  0 | 3  5.1 | 0  0 | 3**q  5.1 |
|  |  | nc | N  % | 12  20.3 | 30  50.8 | 1  1.7 | 43  72.9 | 8  13.6 | 35  59.3 | 0  0 | 43  72.9 | 7  11.9 | 35  59.3 | 1  1.7 | 43  72.9 | 13  22 | 30  50.8 | 0  0 | 43  72.9 |
|  |  | d | N  % | 10  16.9 | 3  5.1 | 0  0 | 13  22 | 8  13.6 | 5  8.5 | 0  0 | 13  22 | 6  10.2 | 7  11.9 | 0  0 | 13  22 | 11  18.6 | 2  3.4 | 0  0 | 13  22 |
|  |  | t | N  % | 22  37.3 | 36  61 | 1  1.7 | 59  100 | 16  27.1 | 43  72.9 | 0  0 | 59  100 | 13  22 | 45  76.3 | 1  1.7 | 59  100 | 24  40.7 | 35  59.3 | 0  0 | 59  100 |
|  | OQ -45  Social roles | i | N  % | 0  0 | 1  1.7 | 0  0 | 1 c  1.7 | 0  0 | 1  1.7 | 0  0 | 1 *h  1.7 | 0  0 | 1  1.7 | 0  0 | 1 m  1.7 | 0  0 | 1  1.7 | 0  0 | 1 r  1.7 |
|  |  | nc | N  % | 13  22 | 28  47.5 | 1  1.7 | 42  71.2 | 8  13.6 | 34  57.6 | 0  0 | 42  71.2 | 6  10.2 | 35  59.3 | 1  1.7 | 42  71.2 | 14  23.7 | 28  47.5 | 0  0 | 42  72.1 |
|  |  | d | N  % | 9  15.3 | 7  11.9 | 0  0 | 16  27.1 | 8  13.6 | 8  13.6 | 0  0 | 16  27.1 | 7  11.9 | 9  15.3 | 0  0 | 16  27.1 | 10  16.9 | 6  10.2 | 0  0 | 16  27.1 |
|  |  | t | N  % | 22  37.3 | 36  61 | 1  1.7 | 59  100 | 16  27.1 | 43  72.9 | 0  0 | 59  100 | 13  22 | 45  76.3 | 1  1.7 | 59  100 | 24  40.7 | 35  59.3 | 0  0 | 59  100 |
|  | OQ-45  Total | i | N  % | 0  0 | 3  5.1 | 0  0 | 3**d  5.1 | 0  0 | 3  5.1 | 0  0 | 3**i  5.1 | 0  0 | 3  5.1 | 0  0 | 3 n  5.1 | 0  0 | 3  5.1 | 0  0 | 3 **s  5.1 |
|  |  | nc | N  % | 2  3.4 | 19  32.2 | 0  0 | 21  35.6 | 1  1.7 | 20  33.9 | 0  0 | 21  35.6 | 2  3.4 | 19  32.2 | 0  0 | 21  35.6 | 3  5 | 18  30.3 | 0  0 | 21  35.6 |
|  |  | d | N  % | 20  33.9 | 14  23.7 | 1  1.7 | 35  59.3 | 15  25.4 | 20  33.9 | 0  0 | 35  59.3 | 11  18.6 | 23  39 | 1  1.7 | 35  59.3 | 21  35.6 | 14  23.7 | 0  0 | 35  59.3 |
|  |  | t | N  % | 22  37.3 | 36  61 | 1  1.7 | 59  100 | 16  27.1 | 43  72.9 | 0  0 | 59  100 | 13  22 | 45  76.3 | 1  1.7 | 59  100 | 24  40.7 | 35  59.3 | 0  0 | 59  100 |
|  | IDS-SR | i | N  % | 0  0 | 3  5 | 0  0 | 3**e  5 | 0  0 | 3  5 | 0  0 | 3 **j  5 | 0  0 | 3  5.0 | 0  0 | 3 o  5 | 0  0 | 3  5 | 0  0 | 3 **t  5 |
|  |  | nc | N  % | 0  0 | 18  30 | 0  0 | 18  30 | 0  0 | 18  30 | 0  0 | 18  30 | 2  3.3 | 15  25 | 1  1.7 | 18  30 | 0  0 | 18  30 | 0  0 | 18  30 |
|  |  | d | N  % | 23  38.3 | 15  25 | 1  1.7 | 39  65 | 16  26.7 | 23  38.3 | 0  0 | 39  65 | 11  18.3 | 28  46.7 | 0  0 | 39  65 | 24  40 | 15  25 | 0  0 | 39  65 |
|  |  | t | N  % | 23  38.3 | 36  60 | 1  1.7 | 59  100 | 16  26.7 | 44  73.3 | 0  0 | 59  100 | 13  21.7 | 46  76.7 | 1  1.7 | 59  100 | 24  40 | 36  60 | 0  0 | 59  100 |

Note. i=increase, nc=no change, d=decrease, t=total

**a.Fisher’s exact test: p=.000, Tau-b=-.57; **b.Fisher’s exact test: p=.004, Tau-b=-.43; c. Fisher’s exact test: p=.272, Tau-b=-.25; **d. Fisher’s exact test: p=.000, Tau-b=-.45; **e.Fisher’s exact test: p=.000, Tau-b=-.52; **f.Fisher’s exact test: p=.001, Tau-c=-.40;**g.Fisher’s exact test: p=.008, Tau-c=-.33;*h.Fisher’s exact test: p=.043, Tau-c=-.26;**i.Fisher’s exact test: p=.001, Tau-c=-.38;**j.Fisher’s exact test: p=.001, Tau-c=-37;** k.Fisher’s exact test: p=.132, Tau-b=-.25; **l.Fisher’s exact test: p=.150, Tau-b=-.31; m.Fisher’s exact test: p=.092, Tau-b=-.32; n.Fisher’s exact test: p=.189, Tau-b=-.23; o.Fisher’s exact test: p=.235, Tau-b=-.24; p.Fisher’s exact test: p=.000, Tau-c=-.50; q.Fisher’s exact test: p=.000, Tau-c=-.43; **r.Fisher’s exact test: p=.072, Tau-c=-.25; **s.Fisher’s exact test: p=.001, Tau-c=-.47; t.Fisher’s exact test: p=.000, Tau-c=-.56;

**Supplementary file 3: scatterplots correlations psychopathology (OQ-45)/depressive symptoms (IDS-SR) and well-being (MHC-SF) at baseline (T1) and follow-up (after 6 months, T2)**


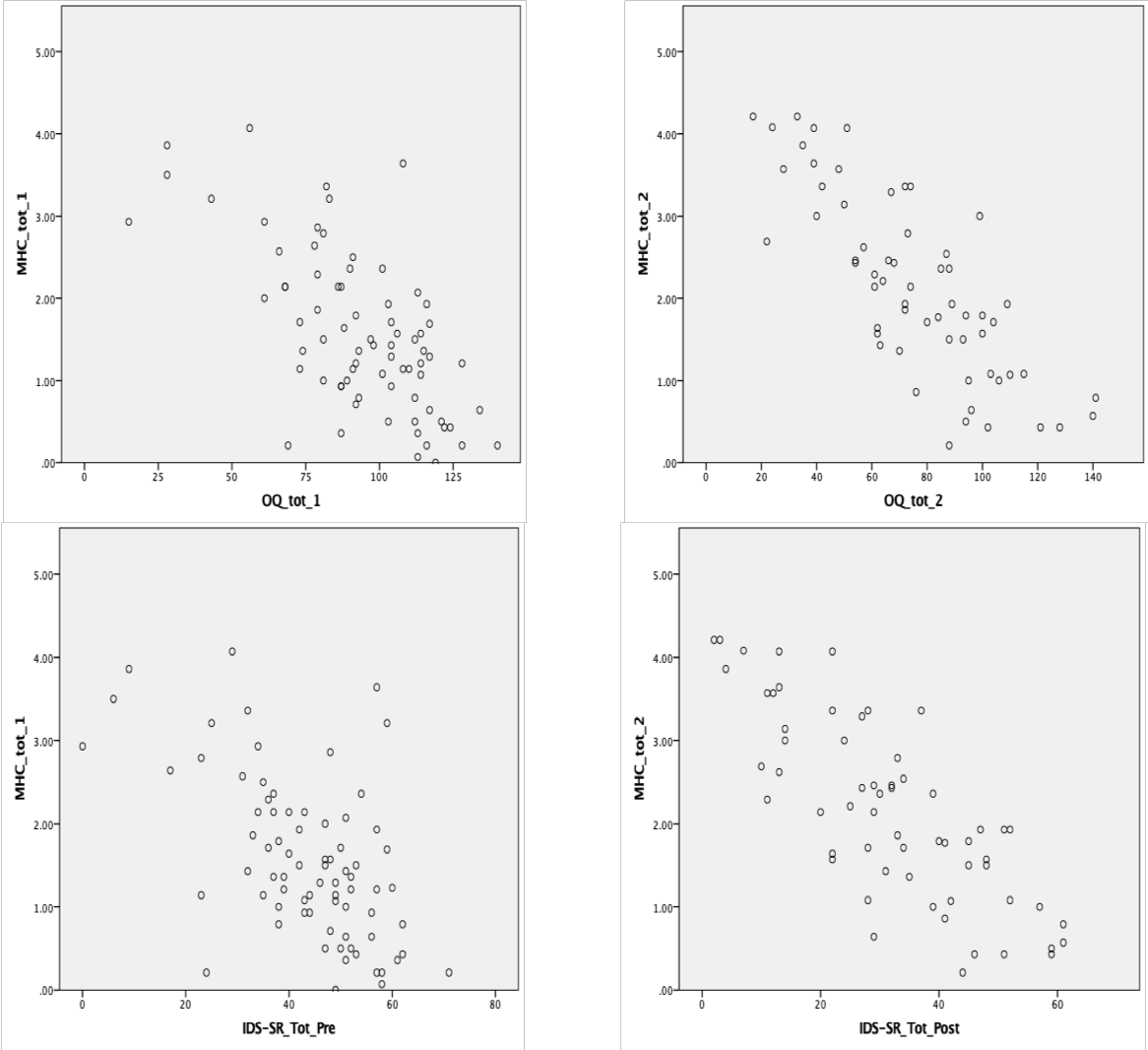


Figure 1. Scatter plots correlations OQ-45 and MHC-SF and IDS-SR and MHC-SF at baseline and follow up
